# Impact of parental leave system on the childbirth plan among working married women: a three-year follow-up study of the Korean longitudinal survey of women and families

**DOI:** 10.1186/s12884-024-06286-5

**Published:** 2024-02-01

**Authors:** Yun Hwa Jung, Yun Seo Jang, Eun-Cheol Park

**Affiliations:** 1 Department of Public Health, Graduate School, Yonsei University, Seoul, Republic of Korea; 2 Institute of Health Services Research, Yonsei University, Seoul, Republic of Korea; 3 Department of Preventive Medicine & Institute of Health Services Research, Yonsei University College of Medicine, 50 Yonsei-ro, Seodaemun-gu, Seoul, 03722 Republic of Korea

**Keywords:** Parental leave, Parturition, Women, Occupational groups, Longitudinal studies

## Abstract

**Background:**

The Korean government seeks to balance work and family and alleviate low fertility by implementing a parental leave system. This study aimed to identify the impact of the parental leave system on childbirth among married working women in South Korea.

**Methods:**

This study used three-year follow-up data from the Korean Longitudinal Survey of Women and Families (2016, 2018, and 2020). The number of participants was 324 at baseline. Logistic regressions using a generalized estimating equation model were performed to examine the impact of parental leave on childbirth. Sub-analyses of covariates, childbirth support, and parental leave systems were conducted.

**Results:**

Of workers covered by the parental leave system, 31.7% considered childbirth. Women covered by parental leave were 3.63 times more likely to plan childbirth (95% confidence interval [CI], 1.32–9.99). The tendency to plan childbirth was pronounced among those in their early 30s (adjusted odds ratio [AOR], 7.20) and those who thought that having children was necessary (AOR, 4.30). Child planning was more influenced by leave support (AOR, 6.61) than subsidies.

**Conclusions:**

Parental leave systems can have a positive impact on working married women’s childbirth plans. Although this system was effective in a group interested in childbirth, it did not create a fundamental child plan. Time support is more important than money concerning childbirth plans. The parental leave system had an impact on childbirth plan. Appropriate parenting policies can effectively increase the fertility rate.

## Background

With a total fertility rate (TFR) of 0.81, South Korea has the lowest fertility rate among member countries of the Organization for Economic Cooperation and Development since 2013 [1]. Multiple European countries, including Spain, Italy, Greece, and the Czech Republic, reached their lowest-low fertility levels in the 1990s. Multiple industrialized countries in East Asia, including Japan, Taiwan, Singapore, and South Korea, followed suit a decade later [2].

The TFR required to ensure a broadly stable population, assuming no net migration and unchanged mortality, is 2.10 children per woman—this is a major social problem. Low fertility rates lead to population decline and rapid aging. This phenomenon exerts pressure on government finances, the health care system, and social divisions and causes discrimination against certain populations [3]. In response to the rapid decline in fertility rate, the Korean government started to promote fertility by adopting various policies since the mid-2000s [4].

Over the last half-century, the participation of women in the labor market has increased drastically [5]. Considering this, the government enacted several policies, including the parental leave policy [6]. Parental leave for up to 1 year (including 90 days of maternity leave) was introduced in 1987 for parents with children under the age of 1 year. The age limit of the children increased to 3 years in 2001 and to 6 years in 2010 to encompass preschool-age children. The maximum length of parental leave is 1 year per child, and this leave can be taken any time before a child reaches 7 years of age [7].

Fertility intention is an important underlying predictor of fertility behavior [8, 9]. Failure to reach the ideal fertility intention is a common cause of low fertility in modern society [10]. Several countries have recently experienced fertility reversals, but none have returned to the replacement level [2]. Parental leave policies can avoid employment instability, which has a negative impact on fertility intentions [11], by balancing work and family and preventing economic disconnection [12]. Additionally, because the reasons for introducing leave policies include the health of the infant and mother [13, 14], they may have a positive impact on fertility intention. Despite the critical role that fertility intention and parental leave policies play in determining women’s fertility behavior and actual fertility, there are very few studies on these aspects in the South Korean context. Some empirical studies have shown a slightly positive impact of family policies on fertility, whereas others have found no significant evidence [15, 16]. Specifically, maternal or paternal leave policy appears to be beneficial for boosting fertility [17, 18], but no definitive effect has been found and reported [19].

Therefore, this study aimed to understand the impact of the parental leave system on childbirth plan among married working women in South Korea. We hypothesized that the beneficiaries of the parental leave system plan to have children in the future.

## Methods

### Data

We used data from the 2016, 2018, and 2020 waves of the Korean Longitudinal Survey of Women and Families (KLoWF), a biennial national survey conducted by the Korean Women’s Development Institute [20]. The KLoWF is a representative panel survey of family life and economic activity among adult women. This survey provides individual- and household-level statistical data of basic socioeconomic factors, family relations and planning, economic careers, and workplace discrimination [21]. The KLoWF data are anonymized, de-identified, confidential, and publicly available secondary material. Therefore, no approval or prior consent from the Institutional Review Board is required.

### Participants

**Fig. 1:**
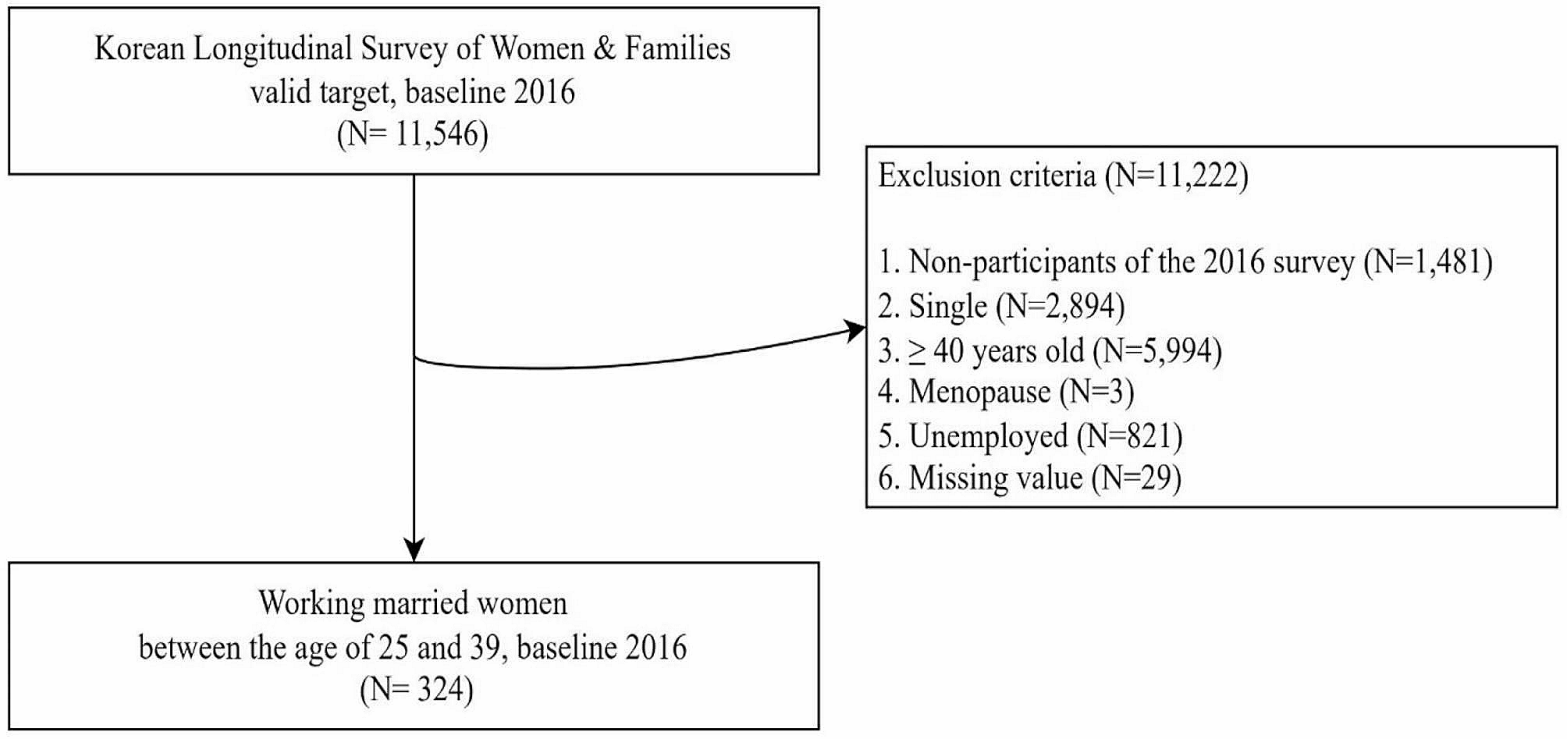
Participant enrollment flow chart

The 2016 baseline study included 11,546 participants, representing 0.05% of middle-aged women nationwide, and this was our study population. Among them, non-responders (*n* = 1,481), unmarried women (*n* = 2,894), those aged over 40 years (*n* = 5,994), menopausal women (*n* = 3), those unemployed (*n* = 821), and those with missing data (*n* = 29) were excluded. A total of 324 married working women aged 25 to 39 were included at baseline (Fig. 1). The following numbers of participants were analyzed in each year: 324 in 2016 (sixth wave), 345 in 2018 (seventh wave), and 227 in 2020 (eighth wave).

### Variables

Application of the parental leave system was the variable of interest. The KLoWF assessed the application of the parental leave system through the question, “Did you receive or can you receive parental leave benefits at work?” We classified “received or could receive” as “applicable” and “not received or could not receive” as “not applicable” and excluded “don’t know.”

Childbirth plan was the dependent variable. In the questionnaire, those who answered “yes” to the question, “Are you planning to have children?” were classified as participants with a childbirth plan. The remaining participants were classified as having no plans for childbirth.

The following covariates were included: demographic variables (age), socioeconomic variables (region, educational level, household income, and occupational categories), family-related variables (other childbirth support systems, view of life for children, housework satisfaction, and current number of children), and health-related variables (subjective health status and stress).

### Statistical analysis

The frequency and proportion of baseline characteristics according to childbirth plan were analyzed using χ^2^ tests. Logistic regression analyses were performed using a general estimating equation (GEE) model to evaluate the impact of the parental leave system on childbirth plans. The main analysis of the relationship between the variable of interest and the dependent variable was adjusted for the covariates. The sub-analyses were stratified by age, region, view of life for children, and current number of children. In addition, we analyzed childbirth plans according to parental leave systems with other childbirth support systems, and parental leave application status which was analyzed over time using lag procedure. All logistic regression analyses were weighted using adjusted odds ratios (AOR) and 95% confidence intervals (95% CI). Results were considered statistically significant at *P* ≤ 0.05. Statistical analysis was performed using SAS, version 9.4 (SAS Institute Inc., Cary, NC, USA).

## Results

The primary analysis included descriptive statistics of the respondents’ general characteristics according to the childbirth plan. Of the 324 participants, 52 (16.0%) considered childbirths. The age of the participants ranged from 25 to 39 years (mean, 35.8 ± 2.9 years). Moreover, those who thought that they needed more children (17.4%), had other childcare support systems (32.5%), and with no current child (74.1%) also planned for childbirth plan (Table 1).

**Table 1:** Baseline characteristics of the study population (baseline 2016)

Variables	Childbirth plan						
	Total		No		Yes		*P*-value
	*N*	%	*N*	%	*N*	%	
**Parental leave system**							< 0.0001
Applicable	82	25.3	56	68.3	26	31.7	
Not applicable	242	74.7	216	89.3	26	10.7	
**Age (mean: 35.8, SD: 2.9)** ^**a**^							< 0.0001
25–29	17	5.2	9	52.9	8	47.1	
30–34	80	24.7	53	66.3	27	33.8	
35–39	227	70.1	210	92.5	17	7.5	
**Region**							0.0519
Urban	138	42.6	109	79.0	29	21.0	
Rural	186	57.4	163	87.6	23	12.4	
**Educational level**							0.0010
College or over	228	70.4	181	79.4	47	20.6	
High school or below	96	29.6	91	94.8	5	5.2	
**Household income** ^**b**^							0.0591
High	109	33.6	84	77.1	25	22.9	
Mid	113	34.9	98	86.7	15	13.3	
Low	102	31.5	90	88.2	12	11.8	
**Occupational categories** ^**c**^							0.0225
White collar	189	58.3	162	85.7	27	14.3	
Pink collar	31	9.6	22	71.0	9	29.0	
Blue collar	31	9.6	22	71.0	9	29.0	
Self-employed, unpaid workers	73	22.5	66	90.4	7	9.6	
**Other childbirth support systems** ^**d**^							< 0.0001
No	241	74.4	216	89.6	25	10.4	
Yes	83	25.6	56	67.5	27	32.5	
**View of life for children** ^**e**^							0.3280
Necessary	241	74.4	199	82.6	42	17.4	
Optional	83	25.6	73	88.0	10	12.0	
**Housework satisfaction**							0.0001
Satisfaction	168	51.9	128	76.2	40	23.8	
Dissatisfaction	156	48.1	144	92.3	12	7.7	
**Subjective health status**							0.1407
Good	245	75.6	201	82.0	44	18.0	
Bad	79	24.4	71	89.9	8	10.1	
**Stress**							0.7246
Less	259	79.9	216	83.4	43	16.6	
Much	65	20.1	56	86.2	9	13.8	
**Current number of children**							< 0.0001
0	27	8.3	7	25.9	20	74.1	
1	68	21.0	49	72.1	19	27.9	
≥ 2	229	70.7	216	94.3	13	5.7	
**Total (** *N* ** = 324)**	324	100.0	272	84.0	52	16.0	

^a^ SD: Standard Deviation

^b^ Household income was stratified into three categories by year based on the sum of earned income, financial income, real estate income, transfer income, and social insurance receipts

^c^ White, pink and blue collars are wage workers based on the International Standard Classification Occupations codes

^d^ Other childbirth support systems include maternity leave, childbirth incentives, and reduced working hours during the childcare period

^e^ View of life for children indicates whether the participants thought that having children in their lives was necessary or optional

As per the GEE analysis of the factors affecting childbirth plans, women covered by parental leave were 3.63 times more likely to have a childbirth plan (95% CI, 1.32–9.99) than those not covered. Women aged 25–29 years were 7.08 times more likely to plan childbirth (95% CI, 2.29–21.86), and women aged 30–34 years of age were 6.06 times more likely to plan for a child (95% CI, 2.87–12.79) than women aged 35–39 years. Women with no child were 22.82 times more likely to have a childbirth plan (95% CI, 7.57–68.80) than women with two or more children (Table 2).

**Table 2:** Analysis results of general estimating equation model with the childbirth plan ^a^

Variables	Childbirth plan			
	Adjusted OR	95% CI		
**Parental leave system**				
Applicable	3.63	(1.32	–	9.99)
Not applicable	1.00			
**Age**				
25–29	7.08	(2.29	–	21.86)
30–34	6.06	(2.87	–	12.79)
35–39	1.00			
**Region**				
Urban	0.69	(0.32	–	1.49)
Rural	1.00			
**Educational level**				
College or over	5.56	(1.51	–	20.36)
High school or below	1.00			
**Household income** ^**b**^				
High	0.81	(0.27	–	2.41)
Mid	0.75	(0.30	–	1.91)
Low	1.00			
**Occupational categories** ^**c**^				
White collar	0.93	(0.27	–	3.22)
Pink collar	1.08	(0.21	–	5.64)
Blue collar	0.98	(0.19	–	5.04)
Self-employed, unpaid workers	1.00			
**Other childbirth support systems** ^**d**^				
No	0.61	(0.22	–	1.73)
Yes	1.00			
**View of life for children** ^**e**^				
Necessary	2.61	(1.19	–	5.72)
Optional	1.00			
**Housework satisfaction**				
Satisfaction	4.93	(2.42	–	10.05)
Dissatisfaction	1.00			
**Subjective health status**				
Good	1.40	(0.63	–	3.12)
Bad	1.00			
**Stress**				
Less	0.47	(0.17	–	1.30)
Much	1.00			
**Current number of children**				
0	22.82	(7.57	–	68.80)
1	2.53	(1.03	–	6.20)
≥ 2	1.00			

^a^ Adjusted OR and 95% CI are exponential results of Analysis Of GEE Parameter Estimates

^b^ Household income was stratified into three categories by year based on the sum of earned income, financial income, real estate income, transfer income, and social insurance receipts

^c^ White, pink and blue collars are wage workers based on the International Standard Classification Occupations codes

^d^ Other childbirth support systems include maternity leave, childbirth incentives, and reduced working hours during the childcare period

^e^ View of life for children indicates whether the participants thought that having children in their lives was necessary or optional

The first sub-group analysis, stratified by the application status of the parental leave system, showed that women were more likely to plan childbirth when the parental leave system was applied than those that were not applied. There was a prominent tendency for planning childbirths in the early 30s than late 30s (early 30s: AOR, 7.20; 95% CI, 1.84–28.14 vs. late 30s: AOR, 3.10; 95% CI, 1.01–9.48). There was a remarkably greater tendency among urban women workers to plan children than rural ones when parental leave was applied (urban: AOR, 7.99; 95% CI, 1.95–32.74 vs. rural: AOR, 6.91; 95% CI, 1.76–27.12). Additionally, women who felt they needed children were more likely to plan for a child when taking parental leave than women who thought having children was optional (necessary: AOR, 4.30; 95% CI, 1.26–14.72 vs. optional: AOR, 3.12; 95% CI, 0.64–15.11). Women who currently have two or more children were noticeably more likely to plan a childbirth than women who had no children or one child when applying to the parental leave system (two or more children: AOR, 14.81; 95% CI, 2.42–90.60 vs. no child: AOR, 4.51; 95% CI, 0.15–128.83; one child: AOR, 4.29; 95% CI, 0.86–21.38) (Table 3).

**Table 3:** Subgroup analysis of independent variables using the GEE of the childbirth plan with the parental leave system ^a^

Variables	Childbirth plan				
	Parental leave system not applied	Parental leave system applied			
	Adjusted OR	Adjusted OR	95% CI		
**Age**					
25–29	-	-	-	-	-
30–34	1.00	7.20	(1.84	–	28.14)
35–39	1.00	3.10	(1.01	–	9.48)
**Region**					
Urban	1.00	7.99	(1.95	–	32.74)
Rural	1.00	6.91	(1.76	–	27.12)
**View of life for children**					
Necessary	1.00	4.30	(1.26	–	14.72)
Optional	1.00	3.12	(0.64	–	15.11)
**Current number of children**					
0	1.00	4.51	(0.15	–	128.83)
1	1.00	4.29	(0.86		21.38)
≥ 2	1.00	14.81	(2.42	–	90.60)

^a^ Adjusted for demographic, socioeconomic, family-related factors, and health-related factors as potential confounders

The second sub-analysis is the result of the childbirth plan according to the parental leave system and other childbirth support systems. Women were more likely to make a birth plan when maternity leave was applied together (AOR, 6.61; 95% CI, 2.17–20.16) or when childbirth subsidy and maternity leave were applied together (AOR, 4.64; 95% CI, 1.35–15.90) than when childbirth subsidy was applied together (AOR, 3.74; 95% CI, 0.19–71.92) (Table 4).

**Table 4:** The results of subgroup analysis stratified by the childbirth support systems ^a^

Variables	Childbirth plan				
	*N* ^b^	Adjusted OR	95% CI		
**Childbirth support system application type**					
Not applicable	229	1.00			
Applicable to childbirth support systems other than parental leave	13	1.63	(0.34	–	7.76)
Applicable to parental leave only	12	3.67	(1.09	–	12.35)
Applicable to parental leave and childbirth subsidy	3	3.74	(0.19	–	71.92)
Applicable to parental leave and maternity leave	39	6.61	(2.17	–	20.16)
Applicable to parental leave, childbirth subsidy, and maternity leave	28	4.64	(1.35	–	15.90)

^a^ Adjusted for other covariates; age, region, educational level, household income, occupational categories, other childbirth support systems, view of life for children, housework satisfaction, subjective health status, stress, and current number of children

^b^ Number of participants by type of childbirth support system at baseline

As regards child planning according to changes in parental leave applications, the tendency to plan childbirth was as follows: new application (AOR, 8.51; 95% CI, 1.06–67.97) > continued application (AOR, 3.99; 95% CI, 0.23–69.34) > continued non-applied (reference group) > excluded application (AOR, 0.83; 95% CI, 0.06–11.80) (Table 5).

**Table 5:** The results of subgroup analysis stratified by the change of the parental leave systems ^a^

Variables	Childbirth plan			
	Adjusted OR	95% CI		
**Changes in the application of the parental leave system**				
Not a beneficiary →Not a beneficiary	1.00			
A beneficiary →Not a beneficiary	0.83	(0.06	–	11.80)
Not a beneficiary →A beneficiary	8.51	(1.06	–	67.97)
A beneficiary →A beneficiary	3.99	(0.23	–	69.34)

^a^ Adjusted for other covariates; age, region, educational level, household income, occupational categories, other childbirth support systems, view of life for children, housework satisfaction, subjective health status, stress, and current number of children.

## Discussion

This study analyzed self-reported data from the KLoWF to examine the impact of parental leave coverage on childbirth plan among working married women. If parental leave was applied, women were more likely to plan to have children. The tendency to have more children was more pronounced among those in their early 30s, urban women workers, and those who thought having children was necessary or who currently had two or more children. In addition, if parental leave was applied together with maternity leave rather than financial support, it had a greater impact on child planning. Married female workers who received parental leave were significantly more likely to have children.

Parental leave beneficiaries were 3.63 times more likely to consider planning childbirth, which is consistent with the findings of previous studies that reported a positive relationship between family policy and parturition [22, 23]. It has been confirmed that the parental leave system positively affects women’s view of marriage [24] and maternal health [25]. In terms of maternal health, parental leave can contribute to reducing the risk of miscarriage and stillbirth for workers of childbearing age. From an economic and social perspective, maternity workers can balance work and family by applying the parental leave system, preventing career interruption [11, 12]. Policies that contribute to maintaining women’s health and socioeconomic status can not only give women a positive perception of childbirth but also contribute to marital stability.

Most of the reproductive women were in their early 30s, followed by those in their late 30s. This is in line with Korea’s many rankings of age-specific fertility rates (fertility rate per 1,000 women in 2021: 30–34 years old, 76 people; 35–39 years old, 43.5 people; 25–29 years old, 27.5 people) [26]. In Korea, women in their late 20s tend to form new personal relationships related to work and marriage. In their early 30s, they spend their time as newlyweds working for stable economic activity and tend to implement their childbearing plans [27]. Since pregnancy at the age of 35 and older is considered advanced pregnancy [28], biological restrictions may hinder fertility, making it difficult for women to plan for children. In other words, the parental leave system can exert a more amplifying policy effect on women of childbearing age who are more interested in childbirth.

Childbirth planning according to the parental leave system still stood out among groups that considered children essential to life. This may be because parental leave positively affects child planning but does not fundamentally affect the establishment of a new birth plan. The motivation for childbirth among women is affected by a complex interplay of economic, social, psychological values, family composition, and so on [29].

Women may receive different motivations for childbirth planning depending on the number of children they currently have [30]. Working married women with two or more children were more likely to plan childbirth when the parental leave system was applied. The birth of a first child is influenced by social norms [11] or largely depends on a person’s view of life who wants to form and solidify a family [31]. . On the other hand, having multiple children may be more influenced by the environment [30]. Countries with less generous spending on family policies had lower intentions of having multiple children [32]. The parental leave system can be a motivating factor for working married women to plan to have multiple children.

In addition, urban women workers who are subject to parental leave are more likely to plan their children than rural women. Cities have relatively dense maternity and pediatric medical infrastructure, while rural areas lack medical infrastructure for mothers and infants [33–35], resulting in essential medical health gaps. Compared with rural areas with a high proportion of the elderly population, cities have a large number of peer groups for young children to socialize with, and the quantity and quality of play facilities, childcare facilities, and educational facilities for young children are relatively good [36, 37].

When parental leave is combined with other maternity policies, the leave support policy is more effective than financial support policies such as subsidies. It was found that working married women receiving parental leave were more likely to plan childbirth when receiving maternity leave rather than childbirth subsidy. This may be because female workers eligible for parental leave consider freedom of time and space more important than economic factors when planning children. Through customized policies that reflect women’s preferred child planning welfare needs, it will be possible to remove the limiting factors of childbirth motivation and increase the chances of childbirth.

Plans for children according to changes in the application of parental leave were in the following order: new application > continued application > continued non-applied > excluded application. This showed a gradual trend depending on the degree of benefit from the parental leave system. Therefore, it is inferred that the inverse causality between parental leave and child planning is low.

This study has some limitations. First, owing to the insufficient number of participants, we did not analyze birth practice according to birth planning. However, we attempted to compensate for this problem indirectly by including the number of current family members as a covariate. In addition, the current number of family members included not only children but also other members such as parents and siblings. This makes it possible to infer the burden of care comprehensively. The number of current family members was also used when considering household income. Second, this study did not analyze the reasons for wanting or not wanting to have children. In addition, fertility among single women or unemployed married women was not analyzed. Therefore, our results should be interpreted with caution. Third, caution is needed in interpretation because it is unknown whether and to what extent childbirth plans and parental leave systems were considered among the factors that participants considered when looking for a job. Although the extent to which the parental leave system is actually used may vary depending on the size of the workplace, employee status, working atmosphere, etc., employees who have worked for more than 6 months are legally subject to the parental leave system regardless of whether they are temporary or full-time. Self-employed workers, freelancers, artists, platform workers, and specially employed workers had legal restrictions on the parental leave system. Nevertheless, the possibility of reverse causality, including changing job types for parental leave systems and childbirth plans when seeking employment, may not be dominant.

This study contributes to making policies that can alleviate barriers to childbirth motivation when low birth rates are a problem. In addition, this study has high external validity because it used representative longitudinal data surveyed nationwide.

## Conclusions

Married working women were more likely to plan childbirth when the parental leave system was applied at their workplace than when it was not. Women in their early 30s, urban women workers, women who thought that having children was necessary, and who currently had two or more children were likely to plan childbirth in the presence of the parental leave system. When the parental leave system was applied together with other childbirth support systems, the childbirth plan was more significant with maternity leave than with childbirth subsidy. Beneficiaries who have newly applied for parental leave were prominent to plan childbirth. Providing appropriate childcare support policies to working women of childbearing age according to their age, place of residence, values, and preferred welfare benefits can help increase the fertility rate.

## Data Availability

Publicly available datasets were analyzed in this study. These data can be found in the SAS version of the personal data at the following link, and those who wish to use it can download the data after consenting; (https://gsis.kwdi.re.kr/klowf/portal/eng/dataSet/rdssFileEngListPage.do?)

## Abbreviations

AOR: Adjusted odds ratio
CI: Confidence interval
GEE: General estimating equation
KLoWF: Korean Longitudinal Survey of Women and Families
TFR: Total fertility rate
